# Can corneal pannus with trachomatous inflammation – follicular be used in combination as an improved specific clinical sign for current ocular *Chlamydia trachomatis* infection?

**DOI:** 10.1186/s13071-016-1308-9

**Published:** 2016-01-27

**Authors:** Tamsyn Derrick, Martin J. Holland, Eunice Cassama, Rod Markham-David, Meno Nabicassa, Michael Marks, Robin L. Bailey, Anna R. Last

**Affiliations:** Clinical Research Department, Faculty of Infectious and Tropical Diseases, London School of Hygiene and Tropical Medicine, London, UK; Programa Nacional de Saude de Visao, Ministerio de Saude Publica, Bissau, Guinea Bissau

**Keywords:** Trachoma, Pannus, *Chlamydia trachomatis*

## Abstract

**Background:**

Trachoma is a blinding disease caused by conjunctival infection with *Chlamydia trachomatis* (Ct). Mass drug administration (MDA) for trachoma control is administered based on the population prevalence of the clinical sign of *trachomatis inflammation – follicular* (TF). However, the prevalence of TF is often much higher than the prevalence of Ct infection. The addition of a clinical sign specific for current ocular Ct infection to TF could save resources by preventing unnecessary additional rounds of MDA.

**Methods:**

Study participants were aged between 1–9 years and resided on 7 islands of the Bijagos Archipelago, Guinea Bissau. Clinical grades for trachoma and corneal pannus and ocular swab samples were taken from 80 children with TF and from 81 matched controls without clinical evidence of trachoma. Ct infection testing was performed using droplet digital PCR.

**Results:**

New pannus was significantly associated with Ct infection after adjustment for TF (*P* = 0.009, OR = 3.65 (1.4–9.8)). Amongst individuals with TF, individuals with new pannus had significantly more Ct infection than individuals with none or old pannus (75.0 % vs 45.5 %, Chi^2^*P* = 0.01). TF and new pannus together provide a highly specific (91.7 %), but a poorly sensitive (51.9 %) clinical diagnostic test for Ct infection.

**Conclusions:**

As we move towards trachoma elimination it may be desirable to use a combined clinical sign (new pannus in addition to TF) that is highly specific for current ocular Ct infection. This would allow national health systems to obtain a more accurate estimate of Ct population prevalence to inform further need for MDA without the expense of Ct molecular diagnostics, which are currently unaffordable in programmatic contexts.

## Background

Trachoma is the most common infectious cause of blindness worldwide and is initiated by conjunctival infection with *Chlamydia trachomatis* (Ct). Children living in trachoma-endemic communities suffer repeated infections with Ct, which manifests as a follicular conjunctivitis (*trachomatous inflammation – follicular* (TF)), often accompanied by papillary inflammation (*trachomatous inflammation – intense* (TI)). Repeated Ct infections can result in fibrosis and scarring of the tarsal plate, leading to in-turning of the eyelashes (trichasis), abrasion of the cornea and eventually blindness. It is currently estimated that ~21.4 million people have active trachoma [1], many of whom will go on to develop the scarring sequelae that can lead to blindness.

The World Health Organization (WHO) aims to achieve global elimination of trachoma as a public health problem by the year 2020 through implementation of the “SAFE strategy” (Surgery for trichiasis, Antibiotics for Ct infection, Facial cleanliness and Environmental improvements to prevent infection) [2]. When the baseline prevalence of TF in 1–9 year olds (TF_1–9_) is between 10–29 % the WHO recommends that 3 years of annual mass drug administration (MDA) with Azithromycin should be delivered, extending to 5 years when TF_1–9_ is ≥30 %. After 3 or 5 years of MDA, impact surveys should be conducted before deciding whether to continue or stop treatment [3]. MDA should be conducted alongside the complementary S, F and E activities. To meet elimination targets, TF_1–9_ is recommended to be below 5 % at the district level. If a district has TF_1–9_ between 5–10 %, within-district communities should be assessed and treated separately by the same principles and F and E should continue.

The use of TF as a clinical sign for Ct infection can however be misleading. In low prevalence areas there is discordance between the prevalence of TF and Ct infection [4–7], particularly after the introduction of MDA [8–10]. Clearance of TF lags behind the clearance of Ct infection on both individual and population levels [8, 11, 12]. It is thought that other chlamydial species and non-chlamydial bacteria can contribute to the presence of TF in low-prevalence settings, although the impact of these infections on scarring development is unclear [13–17]. These observations have led to the suggestion that unnecessary additional rounds of MDA may be delivered by resource poor national eye health programs. This was illustrated by a recent study in The Gambia, where after one round of MDA no ocular infections with Ct were detected in the 1–9 yr olds in the communities under study. This enabled a premature halt to the three year treatment regime in all communities in that treatment arm [18]. Follow up at 3 years showed that TF and Ct infection prevalence were equivalent between communities where MDA ceased after a single round of treatment and in those that had received MDA for 3 years [18]. There are significant costs associated with molecular testing for Ct however, such that cost savings may only be achieved if testing just a sample of children in each census area or in a sample of census areas [19]. Implementation of a simple low-cost Ct diagnostic assay that can be used in regional health care facilities to obtain Ct infection population prevalence data is desirable, however testing is currently confined to expensive commercial platforms, which without subsidies or donations are not feasible in operational settings.

Alternatively, extra information relating to the presence of ocular Ct infection in the community could be ascertained by the inclusion of additional clinical signs that are more sensitive or specific for current ocular infection with Ct, such as corneal pannus. Pannus is the sub-epithelial neovascularization of the peripheral cornea. Corneal pannus was once one of three cardinal clinical signs considered to be essential for the diagnosis of trachoma (alongside “granulations” (follicles) and “conjunctival scarring”) [20–22], however it has now been discarded in the routine diagnosis of trachoma and was not included in the WHO simplified grading system [23]. It was noted that corneal pannus was rarely seen independently and was more often seen alongside follicles or scarring [20], suggesting that pannus is rarely non-specific and that its accompaniment to the two other signs of trachoma could indicate the presence of Ct infection. In this small-scale study we have revisited the utility of pannus as a diagnostic tool that might increase the concordance of clinical signs and current ocular Ct infection.

## Methods

### Ethical, consent and permissions

The study was conducted in accordance with the declaration of Helsinki and was approved by the Ethics committee of the London School of Hygiene and Tropical Medicine, The Gambian Government/Medical Research Council Unit and the Comité Nacional de Ética em Saúde of Guinea Bissau. Written informed consent was obtained from a parent or guardian prior to a participant’s enrollment in the study.

### Clinical sample collection and study design: case control

Samples were collected from children aged from 1 to 9 years residing on 7 islands of the Bijagos Archipelago, Guinea Bissau, 6 islands of which were trachoma treatment naïve at the time of sample collection. Children were screened for clinical signs of trachoma by a trained clinician using the WHO simplified trachoma grading system [23] and the WHO 1981 FPC trachoma grading system [24]. Individuals presenting with follicular scores of “F2” or “F3” using the FPC system were equivalent to “*Trachomatous Inflammation – Follicular (TF)*” using the simplified system and were defined as “TF cases”. Individuals with no clinical signs of follicles (F0), papillary hypertrophy (P0) or conjunctival scarring (C0) were enrolled as “controls” and were matched to TF cases by age, gender and village. Pannus was graded in all TF cases and controls enrolled in the study as not present, new (active neovascularization) or old (empty or scarred vessels). Macro photographs were taken of the edge of the cornea using a Nikon D60 SLR camera with a VR AF-S micro Nikkon 105 mm 1:2.8G ED lens. In this study we evaluated the use of corneal pannus as a clinical sign for Ct infection using field observed grades, analogous to how it would be implemented by national trachoma control programmes.

Two conjunctival samples were taken from the left upper tarsal conjunctiva of each participant with Dacron swabs (Puritan) using standard methodology [10, 25]. Swabs were stored in RNAlater (Life technologies) and kept in a cold chain in the field before subsequent storage at −80 °C. The two swabs from each individual were pooled for RNA and DNA extraction, which was carried out using Norgen total RNA and DNA purification kits following the manufacturers instructions (Norgen Biotek).

### Molecular testing for Chlamydia trachomatis

Droplet-digital PCR (ddPCR) was used to diagnose Ct infection, as described previously [26, 27] with the following modifications. Forward and reverse primers were used at 0.9 μM and probes were used 0.2 μM. Eight microlitres of sample DNA was added to each 20 μl reaction. An initial diagnostic assay detecting Ct plasmid and human RPP30 endogenous control was carried out on all samples. Ct chromosomal gene *omcB* and plasmid DNA were then quantified in positive samples to determine Ct load. Primer and probe sequences are described elsewhere [27]. Thermal cycling conditions were as follows: 95 °C hold for 10 min, followed by 40 cycles of 95 °C for 10 s and 60 °C for 20 s with a final hold of 98 °C for 12 min. ddPCR was performed on a Bio-Rad QX100 Instrument and data were collected using Quantalife software (Bio-Rad). Copy number was calculated in R using previously published scripts [26].

### Statistical analysis

Data were analysed in R (www.R-project.org). Multivariable logistic regression was performed with Ct infection as a binary outcome and clinical signs of TF and pannus were included as independent variables. Age and gender were included a priori.

## Results

**Fig. 1:**
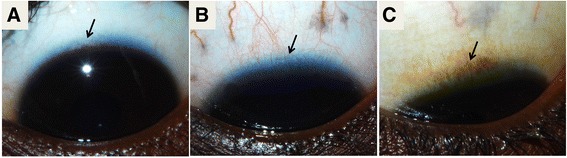
Clinical grading of corneal pannus: example photographs. **a** Normal healthy cornea, **b** new pannus and **c** old pannus. Arrows point to the lack of vascularization in controls **a**, neovascularization in new pannus **b** and old scarred vessels that indicate old pannus **c**

Clinical phenotypes of study participants are shown in Table 1. Eighty individuals had TF, of whom 36 (45 %) had new pannus. Amongst individuals with TF, individuals with new pannus had significantly more Ct infection than individuals with none or old pannus (75.0 % vs 45.5 %, Chi^2^*P* = 0.01). Nine of the 81 controls (11.1 %) and 2 of the 5 Ct positive controls had new pannus. Representative photographs of pannus grades are shown in Fig. 1. Chlamydial load increased with TF grade (data not shown), but there was no apparent relationship between pannus and Ct load (Table 1).

**Table 1:** Clinical phenotypes of study participants

	Controls	TF cases
Total	81	80
% Female	58 %	58.75 %
Median age (IQR^b^)	4 (3–6)	4 (2–6)
Ct positive (%)	5 (6.2 %)	47 (58.8 %)
Ct load^a^ (IQR^b^)	345 (42–1077)	4600 (656–16462)
No pannus	55	34
Ct positive (%)	2 (3.6 %)	15 (44.1 %)
Ct load^a^ (IQR^b^)	36672 (18509–54836)	4500 (1006–6112)
Old pannus	17	10
Ct positive (%)	1 (5.9 %)	5 (50 %)
Ct load^a^ (IQR^b^)	1078	41250 (432–46750)
New pannus	9	36
Ct positive (%)	2 (22 %)	27 (75 %)
Ct load^a^ (IQR^b^)	31 (25–36)	6800 (642–16462)

^a^Ct load = median omcB copies/swab in samples positive for Ct DNA by ddPCR

^b^IQR = Inter-quartile range

In multivariable logistic regression analysis TF was strongly associated with Ct infection (*P* = 1.16 x 10^−7^, OR = 18.17 (6.73–59.5)). New pannus was independently associated with Ct infection after adjustment for TF (*P* = 0.009, OR = 3.65 (1.4–9.8)). Old pannus was not associated with Ct infection (*P* = 0.859, OR = 0.89 (0.23–3.26)) (Table 2). Exclusion of the 31 individuals from the MDA treated island did not significantly change the results of the regression analysis; TF (*P* = 3.83 x 10^−6^, OR = 13.56 (4.83–45.7)) and new pannus (*P* = 0.01, OR = 4.04 (1.43–11.96)) remained significantly associated with current Ct infection (data not shown).

**Table 2:** Multivariable logistic regression of the relationship between clinical phenotypes and Ct infection

	P value	_a_OR^a^ (2.5–97.5 % CI^b^)
TF	1.16 x 10^−7^	18.17 (6.73–59.5)
Pannus: New	0.0088	3.65 (1.4–9.8)
Pannus: Old	0.859	0.89 (0.23–3.26)
Gender (Male)	0.738	0.86 (0.35–2.08)
Age (years)	0.053	1.23 (1–1.52)

Model AIC (Akaike Information Criterion) is 143.46

^a^_**a**_OR: Adjusted Odds Ratio (adjusted for all other variables in the model as listed)

^b^ *CI* Confidence Interval

We investigated the sensitivity and specificity of new pannus and TF as a diagnostic clinical sign for Ct infection. TF was more sensitive than new pannus (90.4 % for TF v 55.8 % for new pannus), whereas new pannus was more specific than TF (69.7 % for TF v 85.3 % for new pannus). The sensitivity of using new pannus and TF together to diagnose Ct infection (excluding individuals presenting with new pannus without TF, or TF without new pannus) was 51.9 % and the specificity was 91.7 % (Table 3). TF and new pannus together had the highest positive predictive value (PPV) (75 %) and a good negative predictive value (NPV) (80 %).

**Table 3:** Performance of TF and new pannus as a diagnostic test for Ct infection

	TF	New pannus	TF & new pannus
Sensitivity	90.4	55.8	51.9
Specificity	69.7	85.3	91.7
PPV	58.8	64.4	75.0
NPV	93.8	80.2	80.0

## Discussion

We have reported the performance of new pannus in combination with TF as a clinical diagnostic test for ocular Ct infection. New pannus was independently associated with Ct infection after adjustment for TF, suggesting that use of the two clinical signs together might increase the likelihood of detecting Ct positive individuals. The presence of TF and new pannus together was a more specific diagnostic test for Ct infection than either alone but it was much less sensitive; therefore use of this combined clinical sign in a trachoma control programme is likely to underestimate the prevalence of Ct infection.

The performance of TF alone as a clinical diagnostic test for Ct infection in these samples was perhaps overinflated by the study design. A population-based survey carried out on four other treatment-naïve islands of the Bijagos Archipelago in Guinea Bissau in 2012 found that in 1–9 year olds, the prevalence of TF was 22 % and the prevalence of Ct infection was 25 % [25]. This present study had a case–control design and participants were enrolled on the basis of clearly defined clinical phenotypes, therefore the likelihood of individuals with TF having Ct infection was high. These are limitations of this study and in order to assess the use of new pannus and TF in combination as a proxy for current Ct infection in an elimination context this experiment should be repeated in a larger population-based survey in a low-prevalence area (TF_1–9_ between 5–10 %). It is possible that in a low-prevalence area the sensitivity of TF and new pannus together as a diagnostic tool for Ct infection would be improved relative to TF alone.

## Conclusions

In communities where the prevalence of TF and Ct infection are known or suspected to be discordant, it may be desirable to use a more specific clinical diagnostic sign for Ct infection in order to prevent an overestimation of the disease burden. The poor sensitivity of the test however renders it inappropriate in a programmatic context. There were significantly more Ct positive individuals with both TF and new pannus compared to individuals with TF alone, therefore a cost-efficient approach for assessing whether to prematurely stop MDA in a community might be to perform molecular Ct testing only on samples from individuals presenting with both clinical signs. Early implementation of the ‘stopping rule’ based on testing a sample of individuals most likely to have Ct infection, or, as we move towards elimination, treating just this sample of individuals most likely to have Ct infection, could lead to significant cost-savings without loss of treatment impact.

### Consent to publish

Written informed consent was obtained to publish anonymized individual-level clinical data and images.

## Abbreviations

Ct: *Chlamydia trachomatis*
TF: *Trachomatous inflammation – follicular*
MDA: Mass drug administration
PPV: Positive predictive value
NPV: Negative predictive value
AIC: Akaike information criterion
